# Self esteem among Tunisian women victims of domestic violence

**DOI:** 10.1192/j.eurpsy.2023.2403

**Published:** 2023-07-19

**Authors:** R. Jbir, L. Aribi, I. Chaari, N. Mseddi, N. Halouani, J. Aloulou

**Affiliations:** Hedi Chaker university hospital, Sfax, Tunisia

## Abstract

**Introduction:**

Intimate partner violence is an under recognized problem in our society that is misjudged and often overlooked.

Violence in women has been linked to chronic health, emotional complications, one of which includes low self-esteem

**Objectives:**

To study the prevalence and predictors of low self-esteem among women victims of domestic violence.

**Methods:**

Our study was descriptive and analytical cross-sectional, carried out with women examined in the context of medical expertise, from May until January 2022.

An anonymous survey was asked to these ladies.

The Rosenberg questionnaire was used to assess the self esteem

**Results:**

122 responses was collected.The average age of the assaulted women in our study was 35.66 years(from18 to 64 years) 98.4%were victims of verbal violence, 95,1% of physical violence, 97,5% of psychological violence and 54.7 %of sexual violence.

Self esteem was very low among 43,4% of women ,low among 18,9%,average among 15,6%,high among 15,6% and very high among 6,6%.

Women with children had lower self-esteem (p=0.02).

Low self-esteem were significantly correlated with: the husband cannabis consumption (p=0.01).

The ladies victims of sexual violence such as an unusual type of relationship had lower self-esteem (p=0.01).

Women who were threatened by their spouses had lower self-esteem (0,01).

An history of aggression during pregnancy was a risk factor for low self-esteem (p=0, 01).

**Conclusions:**

Results suggest domestic violence has on women, not only physically effect but mentally and emotionally, this is why an urgent reaction must be taken by the state to reduce this scourge and hs repercussions on the mental health of the victims.

**Disclosure of Interest:**

None Declared

